# Nasal Cartilage Molding in a Case of Unilateral Cleft Lip and Alveolus (Type D): A Case Report

**DOI:** 10.7759/cureus.50998

**Published:** 2023-12-23

**Authors:** Kushal P Taori, Priyanka P Niranjane, Pallavi Daigavane

**Affiliations:** 1 Orthodontics and Dentofacial Orthopaedics, Sharad Pawar Dental College, Datta Meghe Institute of Higher Education and Research, Wardha, IND

**Keywords:** pre-surgical infant orthopedics, sawangi pre-surgical nasal cartilage molder, unilateral complete cleft lip/palate, nasal deformity, pre-surgical nasal cartilage molding

## Abstract

Nasal cartilage is asymmetric in individuals with cleft lips and has a depressed nasal dome and medial and lateral crus of the nose on the affected cleft side which can be corrected before cheiloplasty by taking advantage of circulating maternal estrogen. This case report presents pre-surgical nasal cartilage molding in a patient with unilateral cleft lip and alveolus using the Sawangi Pre-surgical Nasal Cartilage Molder appliance. The appliance is made from 0.8 mm stainless-steel round wire and has three components, namely, a rectangular frame, a force-generating component, and a swan-shaped wire framework with a soft liner for nasal asymmetry correction.

## Introduction

Unilateral cleft lip and palate (UCLP) cases have sizeable variations in their severity and configuration and, in general, are associated with a more severe nasolabial malformation [1]. Such clefts, which lack soft and hard tissues, present notable surgical challenges for achieving structural, functional, and esthetic results. Mild-to-moderate unilateral incomplete cleft lip with/without cleft palate is linked with nasal deformation [1]. The unilateral cleft palate and lip malformation are widely identified by a broad nasal base and discontinued segments of lips on the clefted side. The affected nasal cartilage on the lower lateral side is displaced inferiorly and laterally, which causes a deformed and depressed nasal dome, an extended alar cartilage rim, an obliquely shaped columella, and an overextended nostril tip [2]. To treat the soft tissue nasal deformity in cleft lip and palate and/or alveolus patients, a study for cartilage molding was conducted by Matsuo et al. According to Matsuo et al., the nasal and auricular cartilage cells in the newborn are soft and pliable and deficient in elasticity. Hence, it can be manipulated to have the esthetic corrections pre-surgically. This is possible because nasal and auricular cartilage consists of chondrocytes and intercellular matrix which mainly consist of collagen, elastin, and a proteoglycan aggregate [3]. The pliability of the cartilage largely depends upon proteoglycan aggregate [4]. In the neonatal period, until the age of three to four months postnatally, the intercellular matrix cells and substances are disconnected due to the flush of hyaluronic acid in the proteoglycan aggregate matrix [4,5]. This hyaluronic acid is elevated by circulating maternal estrogen. There are increased estrogen levels in the fetal circulation at the end of pregnancy and steadily decline after birth until three to four months postnatally. At a more molecular level, increased estrogen enhances oxytocin receptor expression, which, in turn, induces prostaglandin E2 for parturition. This relaxes the cartilage cells and matrix, ligaments, and connective tissue cells of the fetus to go through the parturient canal [4]. The same mechanism occurs with chondrocytes in auricular and nasal cartilage [6]. Hence, pre-surgical nasal cartilage molding and columella lengthening should be started early after the third day of birth to utilize the increased estrogen levels and can only be done until three to four months of postnatal life to get more esthetic and promising results [7-9].

A new classification system proposed by Daigavane et al. is based primarily on the lesser and greater segments and their position around each other [9]. Because of the type and intensity of the defect in UCLP cases, this classification was developed and can be individualized according to the treatment needs. According to the classification Type A UCLP cases have sufficient length of lesser and greater halves separated by less than 8 mm, Type B UCLP cases have more than 10 mm of separation between lesser and greater halves, Type C UCLP cases have both halves separated apart by more than 10 mm of the same length and are arranged parallelly, and Type D UCLP cases have greater alveolar segment overlapping or near each other [9]. These closely associated segments are commonly seen in cleft lip and alveolus cases [10]. In such cases, consideration should only be given to nasal molding [2]. Further, the overlapping of the segments in such cases can be self-relieved by the growth of the maxilla [10]. The Sawangi Pre-surgical Nasal Cartilage Molder (PSNM) was developed exclusively for patients with cleft lips and alveolus (Type D) [10]. As these cases remain untreated at the neonatal age, the molding potential of the cartilage at this age is of no advantage. This demands surgical correction for depressed alar cartilage in later age which leads to increased surgical scar postoperatively and chances of relapse [2]. Hence, taking into consideration reducing the surgical scar according to the esthetic point of view and economic concerns the PSNM was designed and developed [10].

## Case presentation

**Figure 1:**
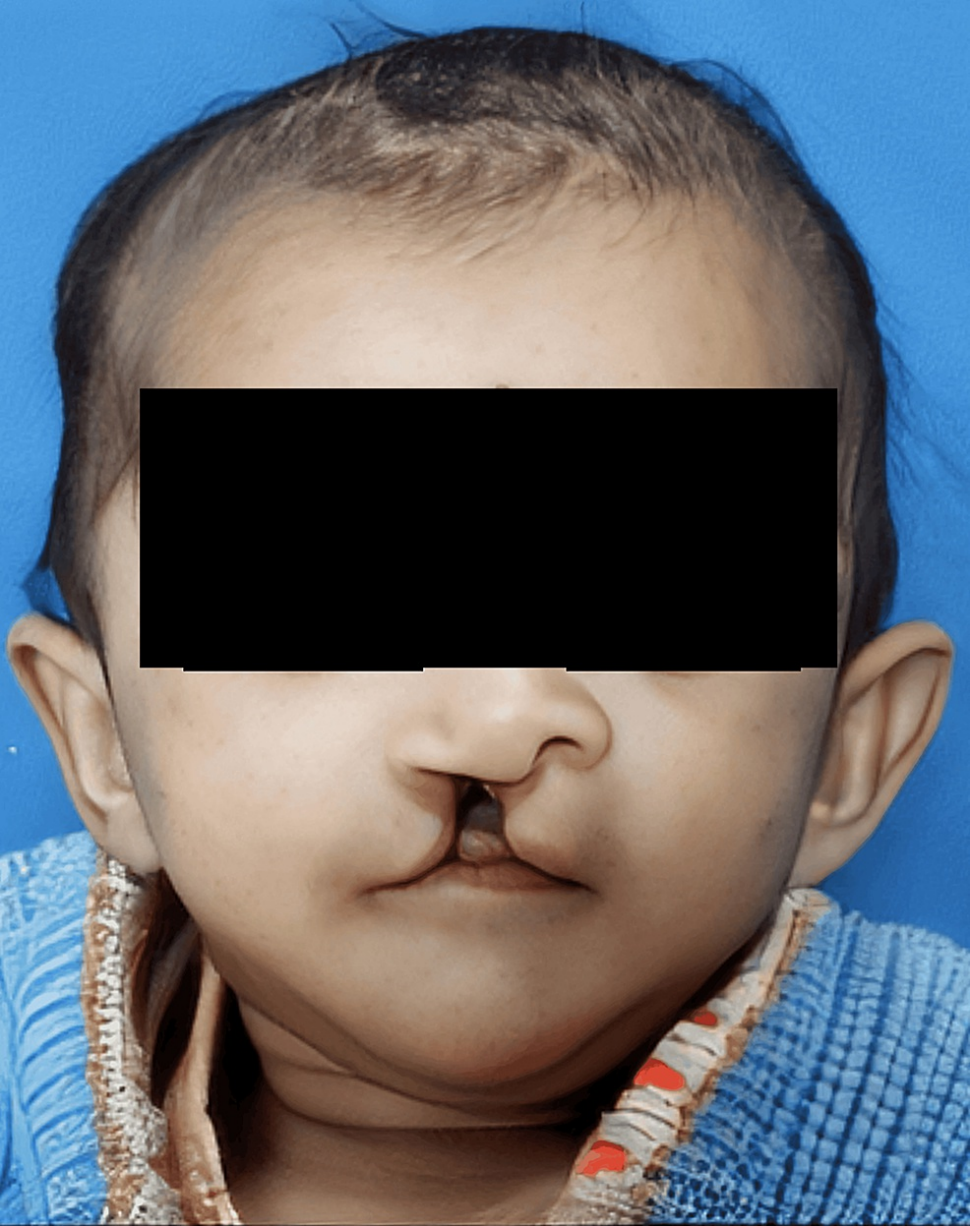
Frontal view showing right-sided cleft lip and alveolus (preoperative).

**Figure 2:**
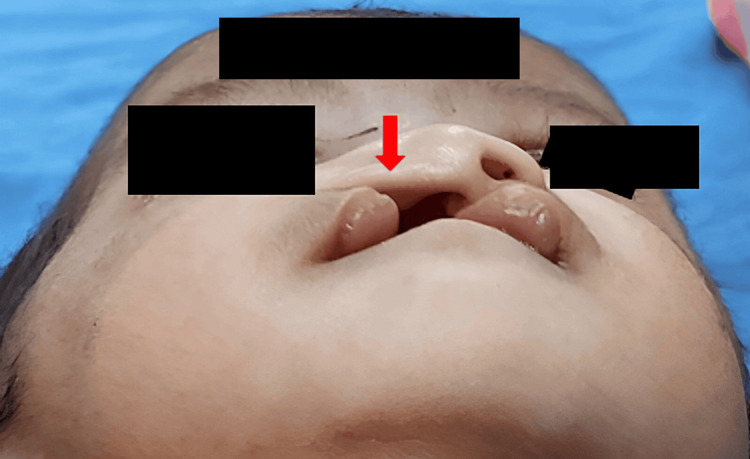
Basal view showing the depressed nasal cartilage with a flat dome (preoperative).

A 22-day-old female patient presented with unilateral right-sided cleft lip and alveolus to Sharad Pawar Dental College and Hospital and was categorized as Type D according to the Daigavane and Hazarey classification [9]. Extraoral examination showed a cleft lip on the right side of approximately 7 mm in width and depressed lower lateral nasal cartilage on the affected side with a depressed or flat dome (Figures 1, 2).

**Figure 3:**
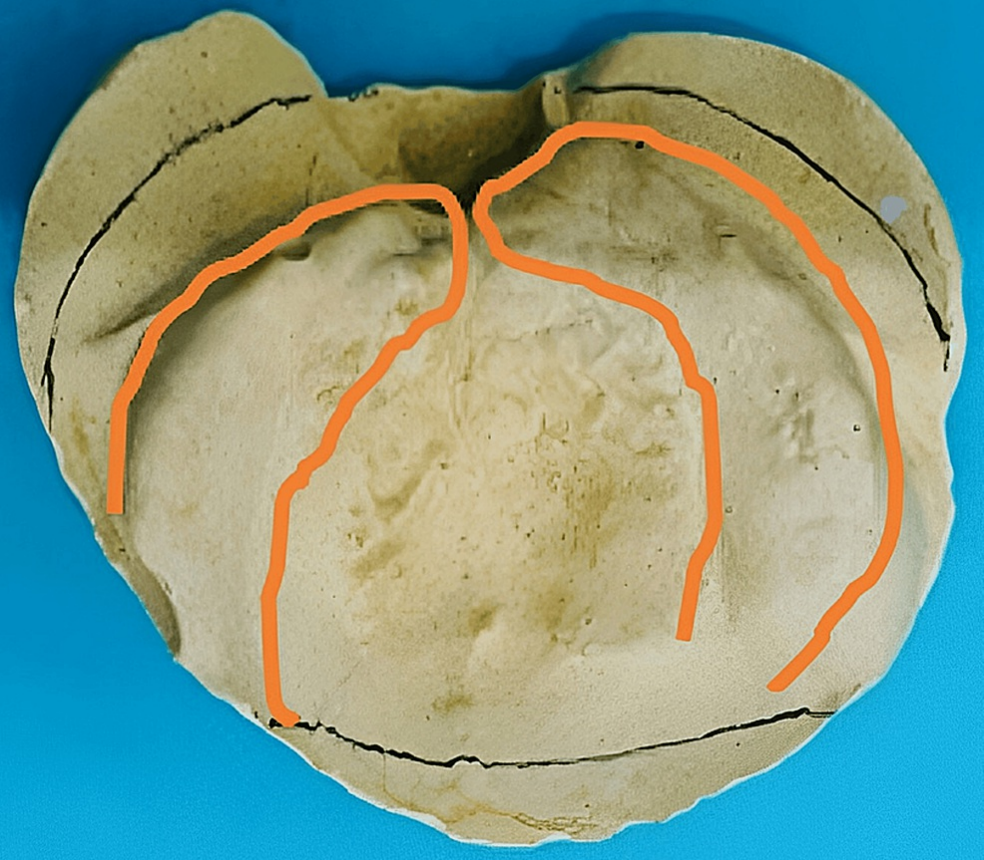
Closely approximated greater and lesser segments in the patient’s cast showing cleft alveolus. Type D according to Daigavane and Hazarey classification.

On intraoral examination, there was no oronasal fistula in the premaxilla and soft palate and there was no history of nasal regurgitation. Greater and lesser segments were very closely approximated with each other and had no cleft on the hard and soft palate (Type D) (Figure 3) [9].

To correct the nasal soft tissue deformity, pre-surgical nasal cartilage molding was planned with PSNM appliance by taking advantage of the plasticity of the nasal cartilage cells.

Sawangi pre-surgical nasal cartilage molding appliance fabrication

**Figure 4:**
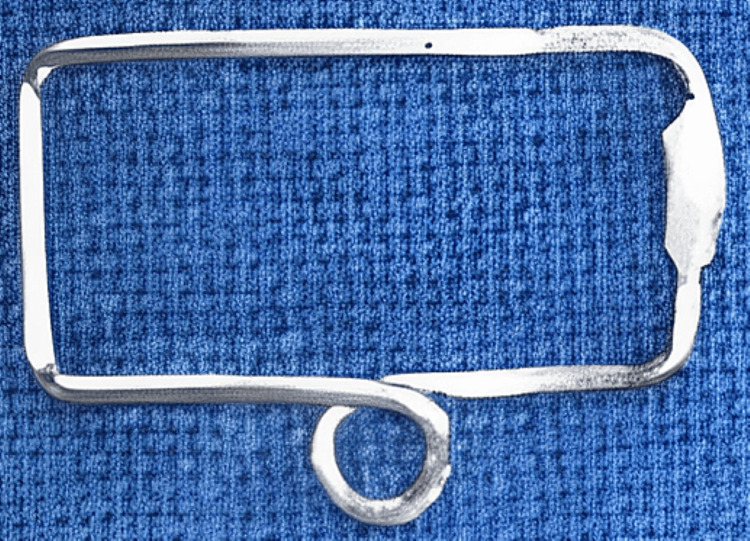
Rectangular framework contoured according to the patient’s forehead and placed at the level of the glabella.

The armamentarium required for the fabrication of the appliance was two surgical micropore tapes of one-inch and a half‑inch diameter, red/yellow elastics, 0.8‑mm stainless steel wire, PermaSoft™ liner, self‑cure acrylic resin, universal hard wire plier and cutter, and soldering unit. The major components of the appliance included a rectangular frame with a helix, a wire framework of an inverted question mark shape with clear acrylic and soft liner bulb for nostril insertion, and an activating component (red/yellow elastics or nickel-titanium close coil spring). A fabricated rectangle framework measuring 10 mm × 15 mm with 0.8‑mm stainless steel wire and helix was incorporated in the lower border for engaging the red elastic. The rectangular frame was contoured as per the forehead of the infant (Figure 4).

**Figure 5:**
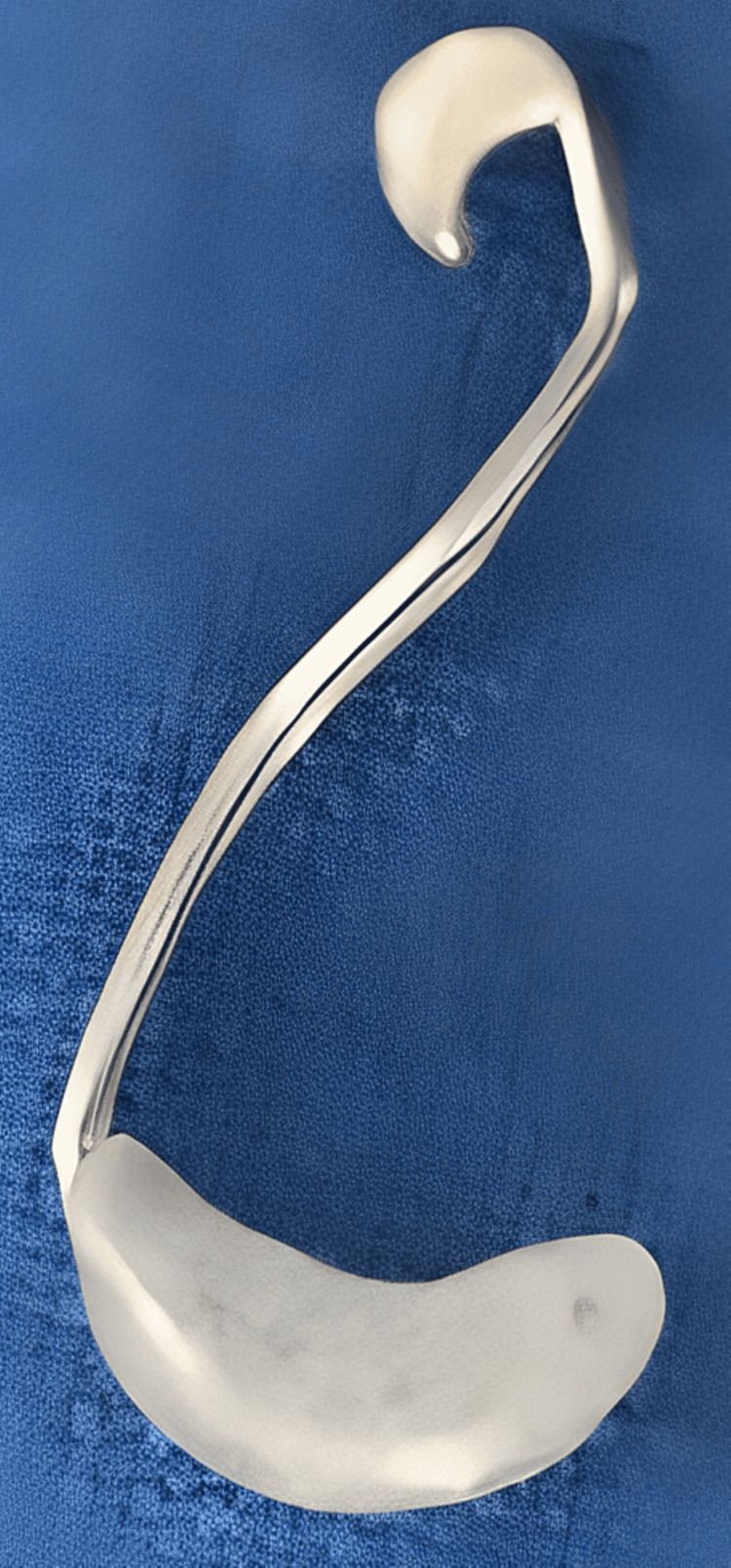
Inverted swan-shaped double-wired framework with an acrylic bulge for retention.

A doubled-wire angular question mark framework was fabricated to be engaged in the nostril (Figure 5).

The distance between the soft tissue glabella and depressed nasal alar cartilage of the cleft nose was measured; the wireframe was fabricated for half of the distance. The tip of the wireframe was lined with self‑cure acrylic and a soft line to fabricate the nasal ball for cartilage molding. The ends of the rectangular frame and wireframe were soldered and smoothened to prevent any trauma.

**Figure 6:**
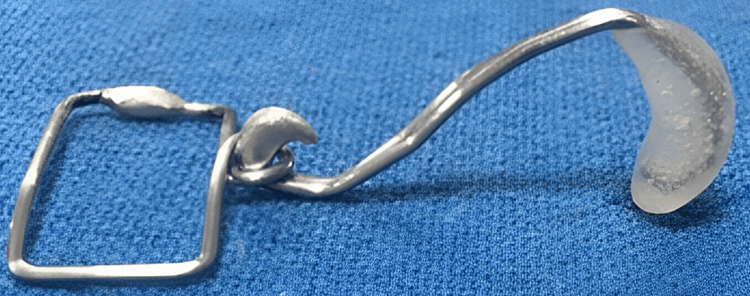
Sawangi Pre-surgical Nasal Cartilage Molding appliance.

**Figure 7:**
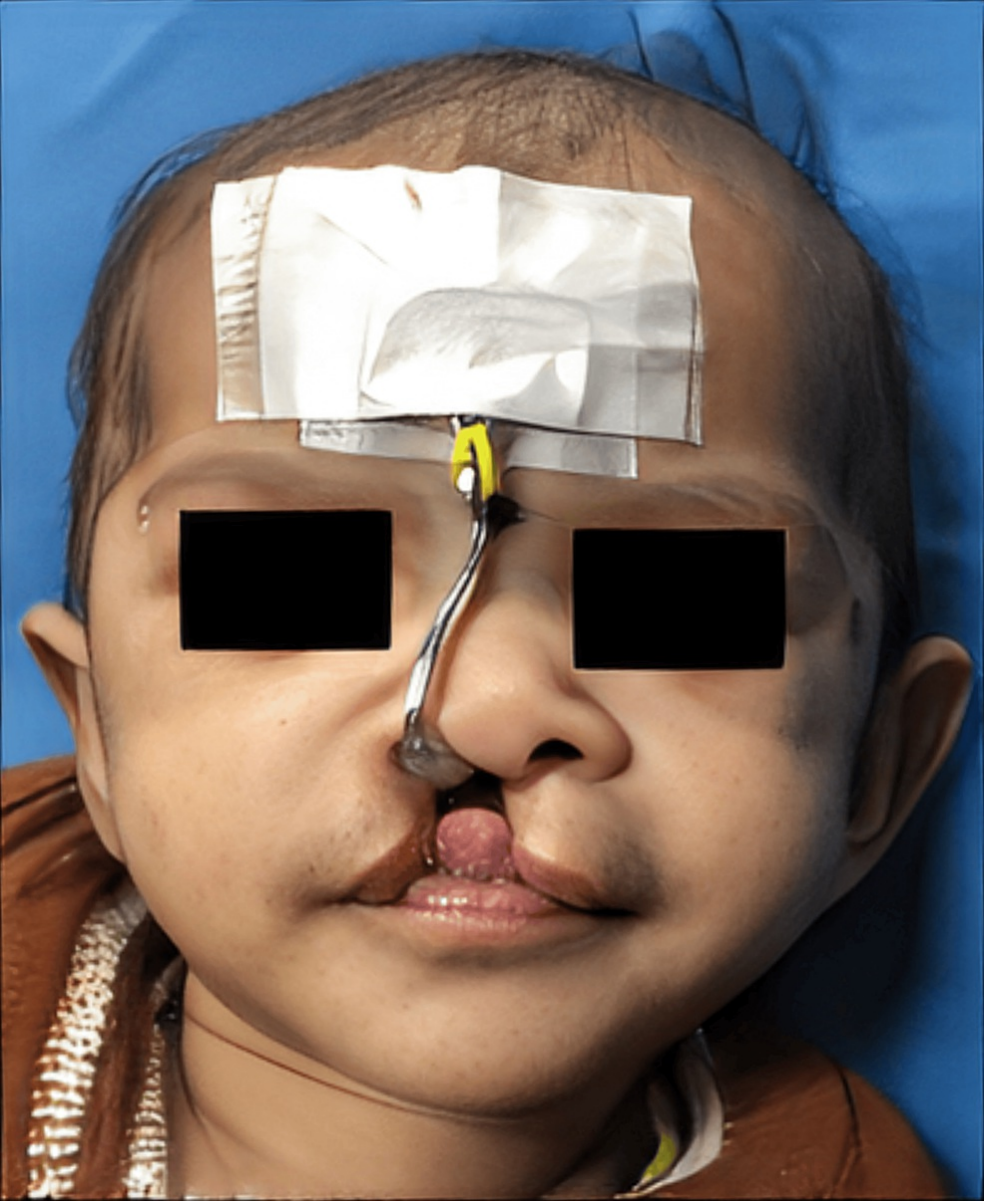
Pre-surgical Sawangi Nasal Corrector appliance in the patient with cleft lip and alveolus.

The rectangular frame was anchored and sandwiched between the surgical micropore tapes on the forehead. One end of the activating component (red/yellow elastic) was engaged on the helix, while the other was on the wireframe hook (Figures 6, 7).

The complete unit was activated by placing the rectangular frame 2-3 mm away from the soft tissue glabella toward the hairline. Parents were instructed to shave the head of the infant before the procedure.

Patient’s outcome

**Figure 8:**
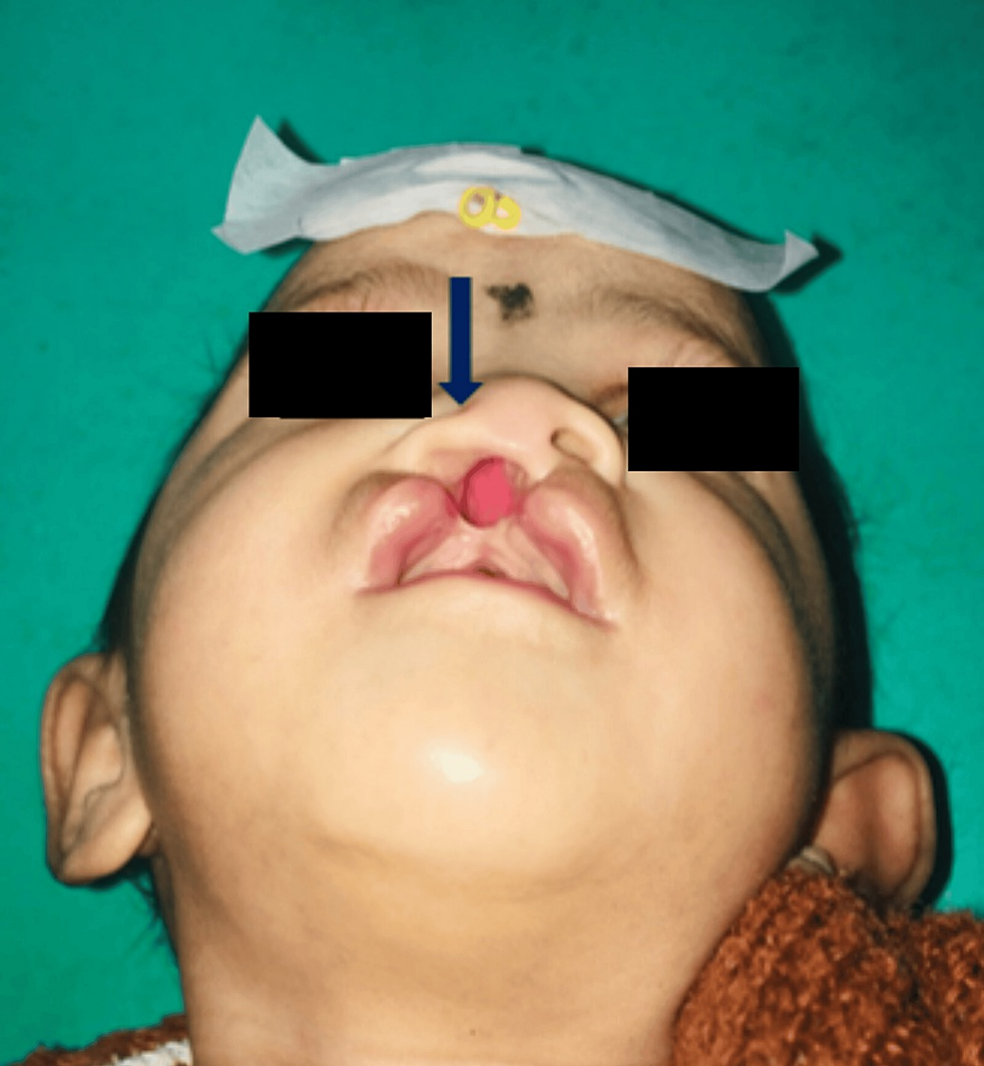
Basal view showing corrections in the depressed alar cartilage (postoperative, pre-surgical).

**Figure 9:**
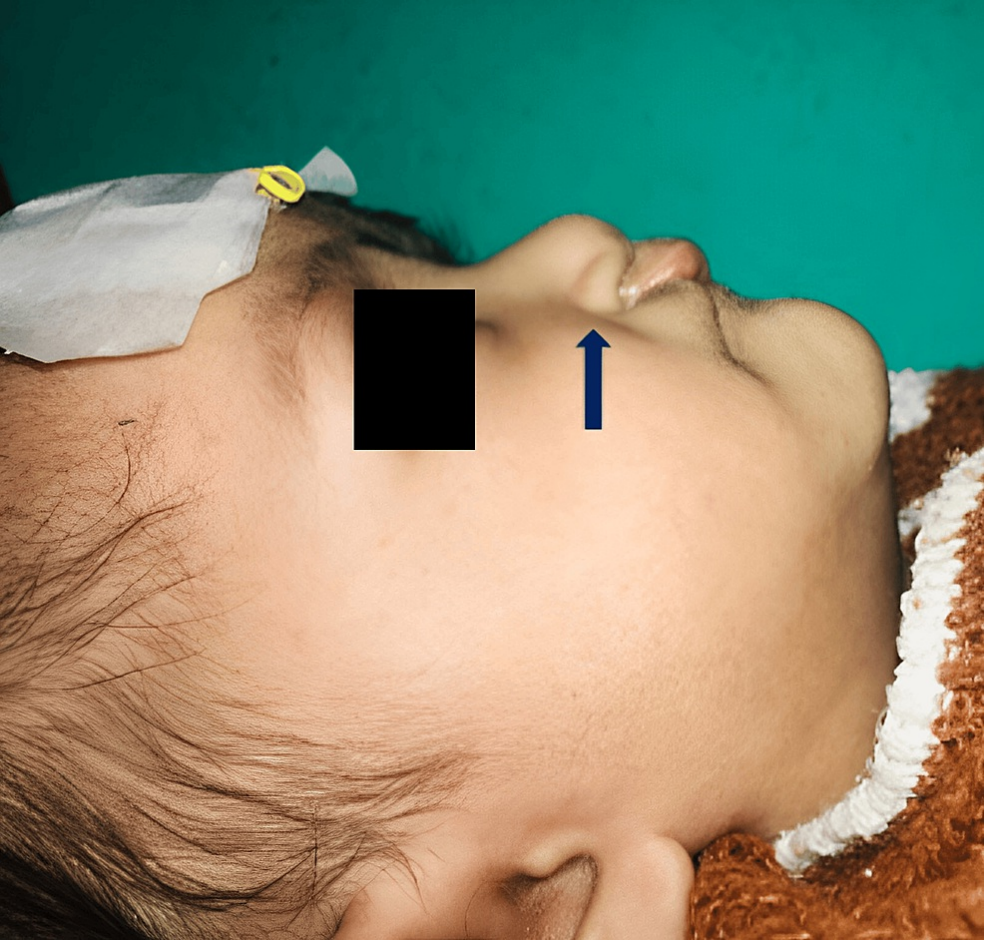
Lateral view showing restored nasal contour (postoperative, pre-surgical).

In the above-treated case of unilateral cleft lip and alveolus case using the PSNM, the nasal depression was elevated within 10 weeks of the active treatment. Columella straightening and lengthening were achieved successfully within the time frame, and facial symmetry was maintained (Figures 8, 9). The period of retention follow-up for the correction achieved was 12 weeks.

## Discussion

The incidence of orofacial clefts is estimated to be approximately 1 in 700 live births [11]. To treat the soft tissue nasal cartilage deformity pre-surgically in cases with cleft lip and alveolus (Type D), before primary lip closure, the PSNM appliance stands tall because of its simple design, cost-effectivity, easy-to-fabricate, and good patient handling, which does not hamper breastfeeding. PSNM should only be employed before three to four months of age because after that the cartilage cells become elastic and resist the change in shape. This occurs due to a steady decrease in the circulating maternal estrogen in the baby’s blood and connective tissue which is advantageous at the time of parturition [12]. Hence, to take advantage, cartilage molding should be started as early as possible [8,9]. In this case, the appliance was employed for 2.5-3 months to correct the nasal cartilage pre-surgically. The appliance was activated until the alar cartilage showed blanching, which signifies the stretch in cartilage cells and intercellular matrix. There are many advantages of the PSNM appliance which include good patient acceptance and economically feasible design; moreover, for the clinician, it is easy to fabricate and requires less chair side time. Furthermore, the appliance significantly altered and improved the cleft nasal deformity. The infant could easily tolerate the appliance. It was comfortable for the patient and parents as they could activate the appliance. Fewer frequent visits to the center were required, and minimum modifications were needed during follow‑up. Moreover, for patients with low socioeconomic status, this was an affordable option.

Liou et al. evaluated the corrective change in the nasal symmetry after nasal molding in UCLP cases based on a three-year follow-up by measuring the quantity of asymmetry in millimeters on standard basilar view photographs [13]. They showed that nasal symmetry improved exceptionally in nasal molding patients followed by cheiloplasty. Hence, stable and acceptable results in nasal symmetry were noted on a three-year follow-up postoperatively [7,13].

## Conclusions

Many clinicians/researchers treat cleft lip and palate cases, but there are very few modalities in the literature to mold the cartilage to bring it back to its normal position non-surgically, thereby reducing scar tissue which is esthetically pleasing and appealing to the patient. Thus, the PSNM was introduced which can be employed in Type D cases from the third day of postnatal life to treat the depressed nasal bridge. Moreover, it is easy to fabricate and activate, is socioeconomically affordable, and has better patient tolerance while breastfeeding as it does not interfere with feeding.
